# Bevacizumab for Newly Diagnosed Ovarian Cancers: Best Candidates Among High-Risk Disease Patients (ICON-7)

**DOI:** 10.1093/jncics/pkaa026

**Published:** 2020-04-04

**Authors:** Olivier Colomban, Michel Tod, Julien Peron, Timothy J Perren, Alexandra Leary, Adrian D Cook, Christophe Sajous, Gilles Freyer, Benoit You

**Affiliations:** p1 Faculté de Médecine Lyon-Sud, Univ Lyon, Université Claude Bernard Lyon 1, EMR UCBL/HCL 3738, Lyon, France; p2 Hospices Civils de Lyon, Pharmacie, Hôpital de la Croix Rousse, Lyon, France; p3 Medical Oncology, Institut de Cancérologie des Hospices Civils de Lyon (IC-HCL), CITOHL, Centre Hospitalier Lyon-Sud, Lyon, France; p4 GINECO-GINEGEPS, Paris, France; p5 St James Institute of Oncology, St James University Hospital, Leeds, UK; p6 Institut Gustave Roussy, Villejuif, France; p7 Medical Research Council Clinical Trials Unit, University College London, London, UK

## Abstract

Bevacizumab is approved as a maintenance treatment in first-line setting in advanced-stage III-IV ovarian cancers, because GOG-0218 and ICON-7 phase III trials demonstrated progression-free survival benefits. However, only the subgroup of patients with high-risk diseases (stage IV, and incompletely resected stage III) derived an overall survival (OS) gain in the ICON-7 trial (4.8 months). The modeled CA-125 elimination rate constant K (KELIM) parameter, based on the longitudinal CA-125 kinetics during the first 100 days of chemotherapy, is a potential indicator of the tumor primary chemo-sensitivity. In the ICON-7 trial dataset, the OS of patients within the low- and high-risk disease groups was assessed according to treatment arms and KELIM. Among the patients with high-risk diseases, those with favorable standardized KELIM of at least 1.0 (n = 214, 46.7%) had no survival benefit from bevacizumab, whereas those with unfavorable KELIM less than 1.0 (n = 244, 53.2%) derived the highest OS benefit (absolute difference = 9.1 months, 2-sided log-rank *P* = .10; Cox hazard ratio = 0.78, 95% confidence interval = 0.58 to 1.04, 2-sided *P* = .09).

In June 2018, the Food and Drug Administration approved bevacizumab in combination with carboplatin and paclitaxel, followed by single-agent bevacizumab, for stage III or IV ovarian carcinoma patients after initial surgical resection (1). This approval is based on the outcomes of 2 parallel phase III trials, GOG-0218 (NCT00262847) and ICON-7 (NCT00483782), which demonstrated benefits in progression-free survival with the addition of bevacizumab to standard first-line chemotherapy in patients with advanced-stage III-IV ovarian cancers (2,3).

However, the best candidate population for adjuvant bevacizumab treatment prescription is still a subject of controversy (4). Indeed, no benefit in overall survival (OS) with bevacizumab was eventually found in the final survival analysis report of GOG-0218 (5). On the other hand, an additional analysis of the ICON-7 trial reported by Oza et al. (6) demonstrated that the specific predefined high-risk population, which included all patients with stage IV and those with unoperated or suboptimally debulked (>1 cm) stage III diseases, derived a benefit in OS with bevacizumab addition: median OS was 39.3 vs 34.5 months and absolute difference 4.8 months (*P* = .03). 

The tumor chemo-sensitivity, as potentially assessed by the modeled kinetics of CA-125 during chemotherapy, might be another parameter to consider. The CA-125 elimination rate constant K (KELIM) is an early modeled kinetic parameter, which can be assimilated to a CA-125 clearance during systemic treatment (7). It is calculated with a minimum of 3 CA-125 values during the first 100 days of neoadjuvant or adjuvant chemotherapy. The prognostic value of KELIM was initially reported in a retrospective study of the CALYPSO phase III trial with recurrent disease patients (7). The prognostic value of KELIM regarding progression-free survival and OS was subsequently validated (when considered as a continuous or categorical covariate) in first-line setting on the data of more than 3030 patients enrolled in 1 phase II and 3 phase III trials, including the ICON-7 trial (8,9).

Here, the objective was to assess the potential complementary prognostic role of KELIM with respect to the Oza et al. (6) risk groups in the ICON-7 trial as a way of defining more accurately the best candidate population for bevacizumab prescription.

The model previously reported (8,9) was used to estimate the modeled KELIM values of patients enrolled in the ICON-7 trial and then to characterize the optimized KELIM cutoff meant to dichotomize KELIM using receiver-operating characteristics. KELIM was standardized (std) by this cutoff (patient std KELIM = patient KELIM:cutoff) and qualified as unfavorable if std KELIM was less than 1.0 or favorable if std KELIM was at least 1.0 as a way of facilitating the clinical interpretation. Survival analyses were performed using univariate log-rank test and Cox model to assess the prognostic value of KELIM with respect to the other prognostic factors within the low- and high-risk patient groups defined by Oza et al., with a landmark time point set up at 100 days. Indeed, the CA-125 kinetics was modeled from day 0 to 100, and exclusion of the early progressions observed during the first 100 days avoided the biases related to the links between early progressions and CA-125 kinetics. All statistical tests were 2-sided, and a *P* value less than .05 was considered statistically significant.

Of 1528 patients enrolled in the ICON-7 trial, the data from 1386 patients (90.7%) could be assessed for survival. The respective median std KELIM were 1.14 days^−1^ (interquartile range = ± 0.58) and 0.96 days^−1^ (interquartile range = ± 0.57) within the low-risk (n = 928) and high-risk (n = 458) disease groups, respectively. The median KELIM value (0.06 days^−1^) was found to be the best cutoff for dichotomizing KELIM. The independent prognostic value of std KELIM, with respect to the other covariates, was confirmed in multivariate analyses (Supplementary Tables 1 and 2, available online). The lack of survival benefit with the addition of bevacizumab in the low-risk group (n = 928) was confirmed regardless of std KELIM value, with excellent median survivals longer than 50 months (Table 1; Figure 1). Among the high-risk disease group patients (n = 458), those with a favorable std KELIM of at least 1.0 (n = 214, 46.7%) did not experience OS benefit from bevacizumab addition (46.6 vs 48.2 months, log-rank *P* = .70, Cox hazard ratio = 0.93, 95% confidence interval [CI] = 0.65 to 1.34). Only those with an unfavorable std KELIM less than 1.0 (n = 244, 53.2%) might have derived a benefit from bevacizumab (median OS = 29.7 months, 95% CI = 24.0 to 35.2 months vs OS = 20.6 months, 95% CI = 17.6 to 23.2 months; absolute difference, 9.1 months; log-rank *P* = .10; Cox hazard ratio = 0.78, 95% CI = 0.58 to 1.04, *P* = .09). The difference was statistically significant when considering noncensored median survivals (Wilcoxon *P* = .004) (Supplementary Table 3, available online). Of note, the survival gain potentially provided by bevacizumab addition was not sufficient to reach similar survivals with those of high-risk disease patients with favorable KELIM (Table 1; Figure 1).

**Table 1.** Outcomes of OS analyses of patients with low- or high-risk diseases according to Oza et al. (6) in ICON-7 trial according to treatment arms, and std KELIM^a^

KELIM category	Treatment arm	Low-risk disease group (n = 928)					High-risk disease group (n = 458)					
		No. ofpatients(events)	Median OS months (95% CI)	Log-rank test	Cox test		No. of patients(events)		Median OS months (95% CI)	Log-rank test	Cox test	
					HR (95% CI)	*P*					HR(95% CI)	*P*
Favorable std KELIM ≥ 1.0	Chemotherapy + bevacizumab	296 (98)	65.0 (61.5 to NR)	0.30	1.18 (0.87 to 1.58)	.28	118 (62)		48.2 (35.4 to NR)	0.70	0.93 (0.65 to 1.34)	.70
	Chemotherapy alone	287 (81)	NR (62.8 to NR)				96 (55)		46.6 (38.4 to NR)			
Unfavorable std KELIM < 1.0	Chemotherapy + bevacizumab	178 (89)	52.8 (36.7 to NR)	0.70	1.05 (0.78 to 1.42)	.75	117 (87)		29.7 (24.0 to 35.2)	0.10	0.78 (0.58 to 1.04)	.09
	Chemotherapy alone	167 (83)	49.8 (39.7 to NR)				127 (96)		20.6 (17.6 to 23.9)			

^a^ All statistical tests were 2-sided. Favorable std KELIM ≥ 1.0 vs unfavorable < 1.0. CI = confidence interval; HR = Cox hazard ratio (chemotherapy alone arm used as reference group); KELIM = CA-125 elimination rate constant K; NR = not reached; OS = overall survival; std = standardized.

**Figure 1.**
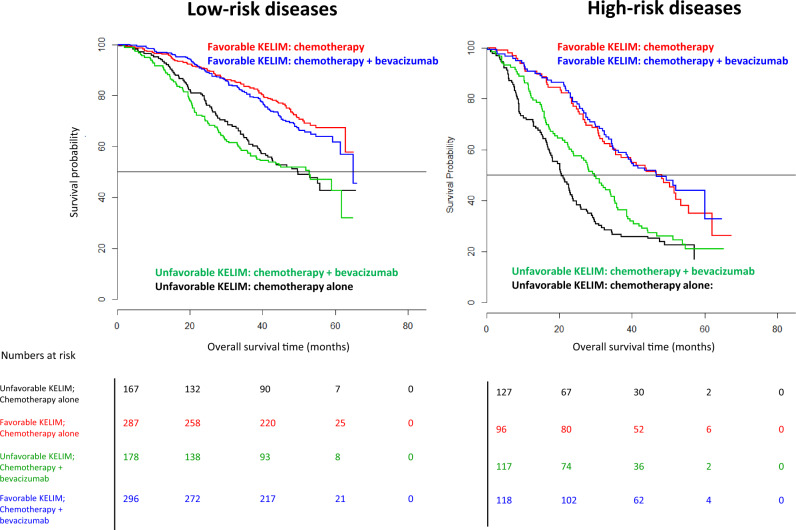
Kaplan-Meier curves of the overall survivals (OSs) of patients with low- or high-risk diseases according to Oza et al. (6) in the ICON-7 trial according to treatment arms, and standardized CA-125 elimination rate constant K (KELIM) (favorable ≥1.0 vs unfavorable <1.0).

Five years after the Oza et al. (6) report, the present additional analysis of ICON-7 trial data suggests that the chemo-sensitivity, as potentially assessed by the modeled CA-125 kinetic parameter KELIM, may be a complementary covariate to consider for decision-making about bevacizumab prescription. Approximately 47% of high-risk patients may not derive survival benefit from the costly addition of bevacizumab, whereas the maximum survival gain (about 9 months) might be obtained in the remaining 53% patients with poorly chemo-sensitive diseases. Such a strategy would imply that all patients would be treated with chemotherapy, and only those with high-risk disease and unfavorable std KELIM <1.0 calculated after 3-4 cycles would receive adjuvant bevacizumab. These data should be interpreted with caution because the limited number of patients in subgroups reduced the power and the statistical significance of the analyses. A validation in other datasets of the hypothesis generated by the present outcomes is necessary.

The recent presentation of PAOLA-1 phase III trial outcomes suggesting a potential benefit from olaparib addition to bevacizumab in patients with incompletely resected stage III and stage IV disease subgroups, contrarily to those of PRIMA and VELIA trials, again raises the question of the best indication of bevacizumab in patients with high-risk diseases (10–12). To be useful in clinics, the KELIM model was implemented on http://www.biomarker-kinetics.org, so any physician can calculate patient KELIM based on their observed CA-125 values (minimum 3 timepoints) during the first 100 days of adjuvant or neoadjuvant chemotherapy.

## Funding

This work was supported by Université Claude Bernard Lyon 1 (France), the employer of OC.

## Notes

**Author contributions: **TJP and ADC were involved in the analytic concept and design and/or participated in the acquisition of the data. OC, MT, JP, AL, CS, GF, and BY contributed to the statistical analyses and participated in interpretation of the results. All authors participated in critical revision of the manuscript for important intellectual content. All authors read and approved the final manuscript.

**Role of the funder:** The funder had no role in the design of the study; the collection, analysis, and interpretation of the data; the writing of the manuscript; and the decision to submit the manuscript for publication.

**Conflicts of interest:** The authors declare no conflict of interest.

**Acknowledgments:** The authors thank all the patients and their families, the investigators, study nurses, pharmacists, pathologists, and all study teams, especially the Gynecologic Cancer InterGroup and MRC Clinical Trial Unit at University College London.

## Supplementary Material

pkaa026_Supplementary_Data

## References

[1] https://www.fda.gov/drugs/resources-information-approved-drugs/fda-approves-bevacizumab-combination-chemotherapy-ovarian-cancer.

[2] Burger RA, Brady MF, Bookman MA, et al Incorporation of bevacizumab in the primary treatment of ovarian cancer. N Engl J Med. 2011;365(26):2473–2483. 22204724 10.1056/NEJMoa1104390

[3] Perren TJ, Swart AM, Pfisterer J, et al A phase 3 trial of bevacizumab in ovarian cancer. N Engl J Med. 2011;365(26):2484–2496. 22204725 10.1056/NEJMoa1103799

[4] Colombo N, Sessa C, Bois AD, et al ESMO-ESGO consensus conference recommendations on ovarian cancer: pathology and molecular biology, early and advanced stages, borderline tumours and recurrent disease. Int J Gynecol Cancer. 2019;29(4):728–760. 10.1136/ijgc-2019-000308 31048403

[5] Tewari KS, Burger RA, Enserro D, et al Final overall survival of a randomized trial of bevacizumab for primary treatment of ovarian cancer. J Clin Oncol. 2019;37(26):2317–2328. JCO1901009. 31216226 10.1200/JCO.19.01009 PMC6879307

[6] Oza AM, Cook AD, Pfisterer J, et al Standard chemotherapy with or without bevacizumab for women with newly diagnosed ovarian cancer (ICON7): overall survival results of a phase 3 randomised trial. Lancet Oncol. 2015;16(8):928–936. 26115797 10.1016/S1470-2045(15)00086-8 PMC4648090

[7] You B, Colomban O, Heywood M, et al The strong prognostic value of KELIM, a model-based parameter from CA 125 kinetics in ovarian cancer: data from CALYPSO trial (a GINECO-GCIG study). Gynecol Oncol. 2013;130(2):289–294. 23694718 10.1016/j.ygyno.2013.05.013

[8] Colomban O, Tod M, Leary A, et al Early modeled longitudinal CA-125 kinetics and survival of ovarian cancer patients: a GINECO AGO MRC CTU Study. Clin Cancer Res. 2019;25(17):5342–5350. 30936122 10.1158/1078-0432.CCR-18-3335

[9] You B, Robelin P, Tod M, et al. CA-125 ELIMination rate constant K (KELIM) Is A Marker Of Chemosensitivity In Patients With Ovarian Cancer: Results from the Phase II CHIVA trial. *Clin Cancer Res.* 2020. DOI: 10.1158/1078-0432.ccr-20-0054. 10.1158/1078-0432.CCR-20-0054 32209570

[10] Coleman RL, Fleming GF, Brady MF, et al Veliparib with first-line chemotherapy and as maintenance therapy in ovarian cancer. N Engl J Med. 2019;381(25):2403–2415. 31562800 10.1056/NEJMoa1909707 PMC6941439

[11] Gonzalez-Martin A, Pothuri B, Vergote I, et al Niraparib in patients with newly diagnosed advanced ovarian cancer. N Engl J Med. 2019;381:2391–2402. 31562799 10.1056/NEJMoa1910962

[12] Ray-Coquard I, Pautier P, Pignata S, et al Phase III PAOLA-1/ENGOT-ov25 trial: olaparib plus bevacizumab (bev) as maintenance therapy in patients (pts) with newly diagnosed, advanced ovarian cancer (OC) treated with platinum-based chemotherapy (PCh) plus bev. Ann Oncol. 2019;30(suppl_5):v894–v934. 10.1093/annonc/mdz3942019.

