# Supplementary material for: Bevacizumab for Newly Diagnosed Ovarian Cancers: Best Candidates Among High-Risk Disease Patients (ICON-7)
Source: JNCI Cancer Spectr. 2020 Apr 4;4(3):pkaa026. doi: 10.1093/jncics/pkaa026 (PMC7306189; doi:10.1093/jncics/pkaa026)
Supplement: pkaa026_Supplementary_Data [file pkaa026_supplementary_data.pdf]

Supplementary Table 1. Outcomes of the prognostic value of Std KELIM and other covariates using univariate and multivariate survival analyses (Log-rank and Cox Proportional Hazard Ratio model) in the whole population.

			Univariate			Multivariate		
Covariates	Classes	N	OS	95% CI	P	HR	95% CI	P
Std KELIM (class)								
Unfavorable	Std KELIM < 1.0	589	34.6	31.0-38.8	<0.001	REF		<0.001
Favorable	Std KELIM ≥ 1.0	797	65.0	61.5-NR		0.49	0.41-0.57	
Arm								
	Chemotherapy alone	677	54.7	49.5-NR	0.98	-		
	Chemotherapy + bevacizumab	709	53.9	49.5-61.8				
FIGO stage								
	I-II	251	NR	NR- NR	<0.001	NS		
	III	957	48.2	44.3-51.7				
	IV	178	32.4	27.5-38.7				
Histology								
	Clear cell	116	61.5	61.5-NR	0.07	-		
	Others	1270	53.1	49.5-59.2				
GCIG CA-125 Response								
	Unfavorable	429	61.5	59.1-NR	<0.01	NS		
	Favorable	936	50.1	47.5-55.8				
Grade								
	1+2	368	53.9	50.3-NR	0.88	-		
	3	998	54.7	49.5-61.8				
High-risk disease								
	No	928	65.0	61.8-NR	<0.001	REF	REF	<0.001
	Yes	458	32.4	29.7-35.5		2.19	1.87-2.56	

Std KELIM (class): standardized KELIM in class; OS : overall survival (months) ; 95% CI : 95% confidence interval; HR : Cox Hazard ratio ; REF : reference ; NR : not reached ; NS : not significant

Supplementary Table 2. Outcomes of the prognostic value of Std KELIM and other covariates using univariate and multivariate survival analyses (Log-rank and Cox Proportional Hazard Ratio model) in the sub-population of high-risk disease patients.

N=458			Univariate			Multivariate		
Covariates	Classes	N	OS	95% CI	P	HR	95% CI	P
Std KELIM (class)								
Unfavorable	Std KELIM < 1	244	23.6	21.3-28.2	<0.001	REF	REF	<0.001
Favorable	Std KELIM ≥ 1	214	46.7	39.6-53.5		0.48	0.38-0.60	
Arm	CP	223	28.9	23.9-33.9	0.09	-	-	-
	CP + Beva	235	35.2	32.4-40.4				
Histology	Clear cell	12	19.4	12.9-NR	0.10	REF	REF	0.05
	Others	446	32.8	29.9-35.8		0.50	0.26-0.99	
GCIG CA-125 Response	Unfavorable	98	31.0	22.8-38.5	0.30	-	-	-
	Favorable	350	32.4	29.7-37.0				
Grade	1+2	115	32.4	29.2-46.6	0.80	-	-	-
	3	335	33.9	27.9-36.9				

Std KELIM (class): standardized KELIM in class; OS: Overall survival (months); 95% CI: 95% confidence interval; REF: reference

Supplementary Table 3. Outcomes of the non-censored overall survival (OS) analyses on dead patients with low-risk or high-risk diseases as per Oza et al., in ICON-7 trial according to treatment arms, and std KELIM (favorable ≥ 1.0, vs unfavorable < 1.0).[4]

N = 651 Death event (735 censored)	Treatment arm	Low risk disease group (Death event, n=351)				High risk disease group (Death event, n=300)			
		Number of Death Event	Median OS (IQR)	Statistics		Number of Death Event	Median OS (IQR)	Statistics	
				Wilcoxon	F			Wilcoxon	F
Favorable std KELIM ≥ 1.0	Chemotherapy + bevacizumab	98	33 (20)	0.57	0.36	62	27 (16)	0.73	0.52
	Chemotherapy alone	81	32 (24)			55	27 (22)		
Unfavorable std KELIM < 1.0	Chemotherapy + bevacizumab	89	21 (16)	0.07	0.95	87	22 (22)	0.004	0.36
	Chemotherapy alone	83	26 (19)			96	17 (15)		

OS: Overall survival in months; IQR: interquartile range; Wilcoxon: Wilcoxon test (P-value); F: homogeneity test of variance (P-value)
